# A Mechanochemical Approach to Porous Silicon Nanoparticles Fabrication

**DOI:** 10.3390/ma4061023

**Published:** 2011-06-07

**Authors:** Luigi Russo, Francesco Colangelo, Raffaele Cioffi, Ilaria Rea, Luca De Stefano

**Affiliations:** 1 Dipartimento per le Tecnologie, Università degli Studi di Napoli “Parthenope”, Centro Direzionale Is. C4 - 80143 Napoli, Italy; E-Mails: l.russo787@gmail.com (L.R.); francesco.colangelo@uniparthenope.it (F.C.); raffaele.cioffi@uniparthenope.it (R.C.); 2 CNR-IMM, Istituto per la Microelettronica e Microsistemi-Unità di Napoli, Via P. Castellino 111, 80131 Napoli, Italy; E-Mail: ilaria.rea@na.imm.cnr.it (I.R.)

**Keywords:** porous silicon, ball milling, nanopowders

## Abstract

Porous silicon samples have been reduced in nanometric particles by a well known industrial mechanical process, the ball grinding in a planetary mill; the process has been extended to crystalline silicon for comparison purposes. The silicon nanoparticles have been studied by X-ray diffraction, infrared spectroscopy, gas porosimetry and transmission electron microscopy. We have estimated crystallites size from about 50 nm for silicon to 12 nm for porous silicon. The specific surface area of the powders analyzed ranges between 100 m^2^/g to 29 m^2^/g depending on the milling time, ranging from 1 to 20 h. Electron microscopy confirms the nanometric size of the particles and reveals a porous structure in the powders obtained by porous silicon samples which has been preserved by the fabrication conditions. Chemical functionalization during the milling process by a siloxane compound has also been demonstrated.

## 1. Introduction

Nanoparticles and nanovectors are being increasingly investigated in industry and academic research due to their widespread applications in all the scientific and production fields. In particular, there is an increasing interest in designing and developing materials suitable as nanocarriers in controlled drug delivery, biological imaging and selective treatment of severe diseases [1,2]. Oncological research asks for multipurpose nanoparticles which can be used, once properly functionalized, against cancer tissues [3]. Many methods to synthesize organic and inorganic nanoparticles have been reviewed, both for a specific aim, such as single molecule detection or intra-cellular imaging, or with many functionalities [3,4]. Among others, silica and silicon nanoparticles have been recently used for *in vitro* and *in vivo* experiments [2]. There is an abundance of top-down and bottom-up processes which can be used to produce silicon and silica nanoparticles: sol-gel synthesis, synthesis in inverse micelles, gas phase reduction, reverse micelles synthesis, pyrolysis or combustion synthesis, laser ablation and sputtering deposition [5,6,7,8]. Also, anodizing silicon wafers in hydrofluoric acid solution, followed by sonication, can be used to fabricate porous silicon (PSi) nanoparticles [2,9,10,11].

Even if some of the cited techniques can give very uniform shaped nanoparticles with size less than 50 nm, in this work we investigated a mechanochemical top-down technique like the ball-milling process as an alternative production method. This technique is already used on an industrial scale in many other fields, such as mechanical alloying [12], hydrocarbons conversion [13,14], dehalogenation [15], solid amorphization [16], drugs synthesis [17] *etc*. All these processes are based on a very effective mechanical energy transfer between the grinding media and the substrate assured by continuous impacts. The induced stresses increase the internal tensions leading the material to a metastable state. As a consequence, the material may release energy in many ways such as fractures, plastic deformations, amorphization, oxidation, crystal defects and radicals formation, so that activation of the surfaces and a strong comminution in size are achieved. A three-stage mechanism has been proposed to explain how to achieve nanometric dimensions from macroscopic samples which involves deformation in shear bands, annihilation and recombination of dislocations, and randomization of crystallites orientation [18]. Milling processes are characterized by mild operating condition (*i.e.,* standard pressure and temperature), and possibility of large-scale production. Moreover, ball milling requires light instrumentation equipment and controls so that it can be classified as an affordable technique for laboratories not specialized in material chemistry. At the same time, there are many different process variables that can be tuned in order to obtain a product with well established properties: milling time, milling speed, size of grinding media, ball-to-powder ratio, controlled atmosphere, and so on. This fabrication method is thus simple but not trivial and it can be an attractive technology in nanopowder production. 

In this work, we have produced and characterized porous silicon nanopowders starting from porous silicon membranes by using a mechanochemical approach which allows a fair control of the powders’ features and also their simultaneous functionalization.

## 2. Experimental Section

In our experiments, we fabricate porous silicon membranes starting from crystalline silicon, available as commercial wafers p^+^-doped, 400 ± 25 μm thick, <100> oriented. Following a well known procedure [19,20], the partial dissolution of the wafers has been obtained in a HF-ethanol aqueous solution. The electrolytic solution was prepared by mixing HF (39.5% wt.) and anhydrous ethanol in 3:7 by volume. The wafer and the solution were placed in a Teflon cell where a current density of 40 mA/cm^2^ was applied for 1 h. A current density peak of 800 mA/cm^2^ has been used to release the porous silicon membranes, which had a mean porosity of 45% and a mean thickness of 120 μm. The thickness and porosity of the membranes have been estimated by spectroscopic reflectivity measurements [21].

The milling experiments were carried out in a FRITSCH Pulverisette 6 planetary ball mill equipped with 80 mL agate vial and grinding balls of 10 mm diameter. The planetary ball mill owes its name to planet-like movements of the vial: the vessel rotates around the mill’s central axis and, at the same time, around its own axis in the opposite direction. The movement of grinding balls inside the vial ensures continuous impact with the material. Millimetric pieces of porous silicon samples and of crystalline silicon, for comparison purposes, previously subjected to a coarse grinding in an agate mortar, were placed in the vial and then sealed by a clamp. Milling conditions are reported in Table 1. After each milling run, we collected the powders and characterized the samples by X-ray powder diffraction (XRPD), Fourier-transform infrared spectroscopy (FT-IR), BET surface analysis and transmission electron microscopy (TEM) in order to get information on crystallites sizes, and their physical and chemical properties.

XRPD analysis was performed by two different Philips instruments (PW1710 diffractometer and PANalytical X’Pert PRO MPD diffractometer), both using Cu Kα radiation (λ = 0.154056 nm). On all the XRD patterns the stripping of Kα2 component of Cu radiation was carried out and the peaks were fitted with pseudo-Voigt function.

Silicon nanopowders were dispersed in KBr by mixing about 1 mg of silicon with 300 mg of KBr, and pelletized in a press. FT-IR spectra were acquired by a JASCO FT/IR-430 spectrometer (64 replicas, 4 cm^−1^ resolution). Porous samples surface analysis was characterized by a QUANTACHROME Autosorb-1 analyzer. Adsorption isotherms were studied and BET analysis obtained the pores size distribution by means of BJH method. 

Samples for TEM analysis were prepared by wetting a minimal fraction of the powders by ethanol in an ultrasonic bath (Elmasonic S10, amplitude 30 W, t = 2 min). Since after this first sonication the samples still appeared agglomerate on TEM images, the powders were treated in a SONICS VIBRA-CELL VCX500 500W (amplitude 50%, t = 2 min). A drop of the suspension was then placed on a copper grid and dried. The sample holder was placed in the TEM microscope: FEI TECNAI G2 SPIRIT TWIN (120 kV, LaB6). TEM images were analyzed by the free software ImageJ (rsbweb.nih.gov/ij/). A fraction of the pSi-2 sample was added by 3-aminopropyltriethoxysilane (APTES). According to the method proposed by some authors [21,22], the powders were firstly oxidized in a 20% v/v nitric acid solution and then milled in 10% by volume APTES solution in anhydrous toluene for 10 min. After rinsing and centrifugation steps, powders were collected and FT-IR analysis was performed.

**Table 1:** Experimental working conditions.

Sample	Substrate	Mass (g)	Ball-to-powder weight ratio	Time (h)
cSi-0.1	monocrystalline	0.399	10:1	0.1
cSi-0.5	monocrystalline	0.570	10:1	0.5
cSi-2	monocrystalline	0.575	10:1	2
cSi-5	monocrystalline	0.561	10:1	5
cSi-10	monocrystalline	0.562	10:1	10
cSi-20	monocrystalline	0.564	10:1	20
cSi-20b	monocrystalline	1.878	20:1	20
cSi-0.1b	monocrystalline	1.497	20:1	0.1
pSi-0.1	porous	0.916	20:1	0.1
pSi-2	porous	0.909	20:1	2
pSi-4	porous	0.900	20:1	4

## 3. Results and Discussion

**Figure 1:**
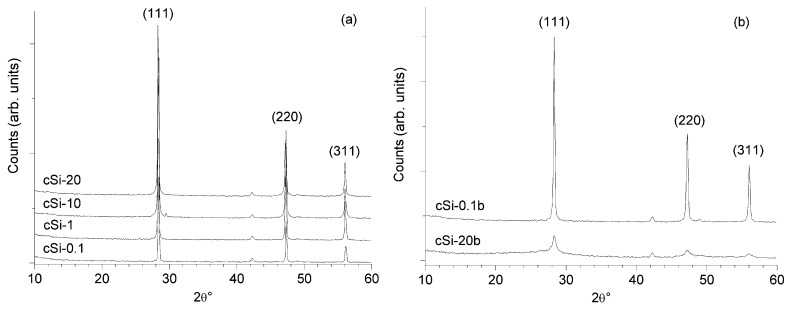
X-ray powder diffraction (XRPD) patterns for crystalline samples: (**a**) samples milled 0.1 h, 1 h, 10 h and 20 h with a 10:1 b/p ratio; (**b**) samples milled 0.1 h and 20 h with a 20:1 b/p ratio.

As a reference characterization technique for the silicon powder produced, we have chosen XRPD since the analysis of the diffraction patterns can be very useful to study the changes of the crystal structure induced by the milling processes. Starting operation conditions have been established on crystalline silicon: a first milling run on crystalline silicon sample in 10:1 ball-to-powder ratio was completely ineffective in size reduction up to 20 hours of treatment as can be noted by the diffraction patterns observed in Figure 1a. In this case, the patterns do not show any change, and we can conclude that the milling process did not affect the material. When the ball-to-powder (b/p) ratio was increased up to 20:1 (Figure 1b), the broadening of the peaks clearly appeared and the intensity decreased due to crystallites sizes dispersion and since the amorphization became significant. Weak signals around 42.2° and 49.1° have been assigned to (111) and (220) characteristic peaks of chromium present as impurity in the instrumentation. It is important to note that even if the running time was so prolonged, we have not found any contamination due to abrasion of the mortar.

**Figure 2:**
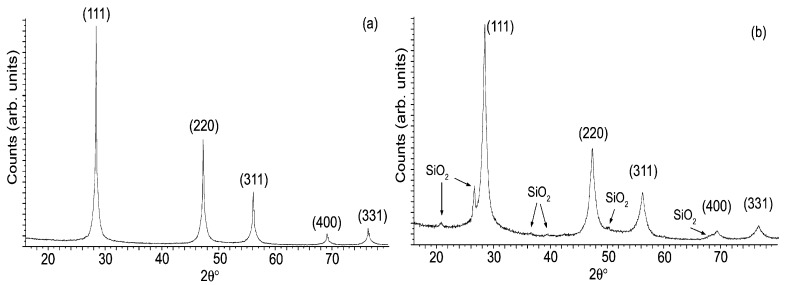
XRPD patterns for porous samples: (**a**) pSi-0.1 sample milled 0.1 h, and (**b**) pSi-2 sample milled 2 h: the SiO_2_ phase is detected.

We thus used the same b/p ratio for the comminution of PSi membranes. Figure 2 shows XRPD patterns of porous samples milled for 0.1 h (a) and 2 h (b). These patterns were collected by the Philips X’Pert Pro diffractometer and by using the software subroutine for Rietveld quantitative analysis we found that there were not any different silicon phases in the pSi-0.1 sample, while an 8.7% of SiO_2_ was identified in the pSi-2 sample. It is well known that crystallites size can be estimated from diffraction pattern analysis by measuring the full width at half maximum (FWHM) measurement and applying the Scherrer equation:

$$d=\frac{K\lambda }{B\cos\theta }$$

where B is the FWHM, K is the Scherrer constant (1 > K > 0.89), λ is the wavelength in nanometers, and d the mean crystallite size [23]. The peak broadening also depends on the lattice strain induced by mechanical stresses, so that the Williamson-Hall method can be used to improve the analysis [11]. Introducing the component for peak broadening due to lattice strain and rearranging Scherrer equation, we obtain:

$$B\cos\theta =\frac{K\lambda }{d}+\eta \sin\theta$$

where the coefficient η represents the lattice strain. By plotting the term *Bcosθ* values for each peak *vs. sinθ* and applying a linear fit, crystallites size d can be obtained from y-intercept. In this case, only a mean value of the crystallite dimension can be obtained, which is an average between all the diffraction peaks generated by the nanopowders.

The crystallites size obtained for crystalline and porous silicon samples are compared in Table 2, when estimated by the Scherrer equation, and in Table 3, when obtained using the Williamson-Hall method: a significant crystallites size decrease trend can be clearly noted on increasing the milling times and, in the case of porous samples, nanometric dimensions are achieved in considerably shorter times.

**Table 2:** Crystallites size obtained by means of Scherrer equation.

Sample	Crystallites size (nm)				
	(111)	(220)	(311)	(400)	(331)
cSi-0.1b	56 ± 1	58 ± 1	42 ± 1		
cSi-20b	16 ± 1	13 ± 1	11 ± 1		
pSi-0.1	36 ± 2	26 ± 4	25 ± 3	22 ± 3	23 ± 3
pSi-2	12 ± 2	10 ± 1	10 ± 1	10 ± 1	8 ± 1

Both theories estimated consistent values for the crystallites sizes within the errors.

**Table 3:** Crystallites size obtained by means of Williamson-Hall plot.

Sample	Kλ/d	η	d (nm)
cSi-0.1b	0.002 ± 0.001	~ 0	62 ± 30
cSi-20b	0.0073 ± 0.0008	0.004 ± 0.002	19 ± 2
pSi-0.1	0.0023 ± 0.0002	0.0064 ± 0.0008	59 ± 6
pSi-2	0.009 ± 0.002	0.011 ± 0.003	15 ± 3

**Figure 3:**
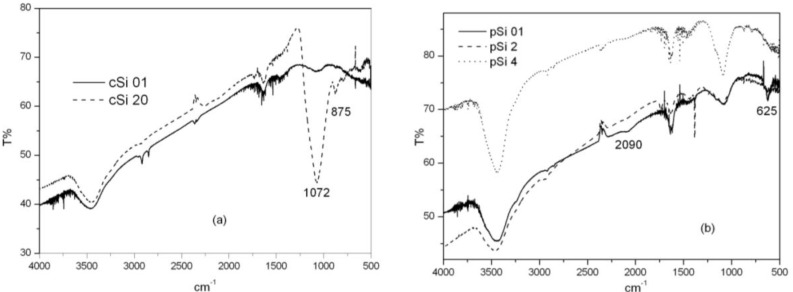
Fourier-transform infrared spectroscopy (FT-IR) spectra for the crystalline samples: (**a**) milled at 0.1 h and 20 h; (**b**) porous samples milled at 0.1 h, 2 h and 4 h.

Infrared absorption analysis reveals information on powders’ surface chemistry and how it changes as a function of the milling times. Figure 3 shows infrared spectra for the crystalline (a) and porous (b) samples milled at different times. Typical Si-O and Si-O-Si bond stretching characteristic peaks around 1080 cm^−1^ and 800 cm^−1^ can be observed which increases with milling times. The position of the 1080 cm^−1^ band depends on the x value of SiO_x_, in this case corresponding to a x value of 1.8. The peak around 875 cm^−1^ has been related to vibrational modes from structural combinations of Si-(O_2_Si_2_), Si-(O_3_Si) and Si-(O_4_) [24]. The porous samples spectra (b) show a new peak around 625 cm^−1^, due to the hydrogenated surface of fresh etched porous silicon, related to Si-H bending mode of the Si_3_-SiH group and also a peak at 2090 cm^−1^ (νSi-H) [25]. The bands disappear when milling time is extended due to the oxidation of Si-H bonds.

Surface area and porosimetric analysis were carried out on porous samples pSi-0.1 and pSi-4. Adsorption measurements of 0.1 h milled sample can be classified, according to IUPAC guidelines, as type IV isotherms with H1 type hysteresis loop (data not shown here) [26]. This adsorption behavior is attributed to spherical agglomerates of porous materials with a narrow pores distribution. From BET analysis a specific surface area of 105.2 m^2^/g is obtained. Moreover, the BJH method reveals a narrow pores size distribution around 6 nm radius.

The adsorption measurement of 4 h milled sample shows a type III isotherm with a small hysteresis loop related to a macroporous material. The BET analysis estimated lower specific surface area equal to 28.7 m^2^/g and a larger dispersion of pore sizes. This is due to partial destruction of the porous skeleton achieved by extended milling time.

**Figure 4:**
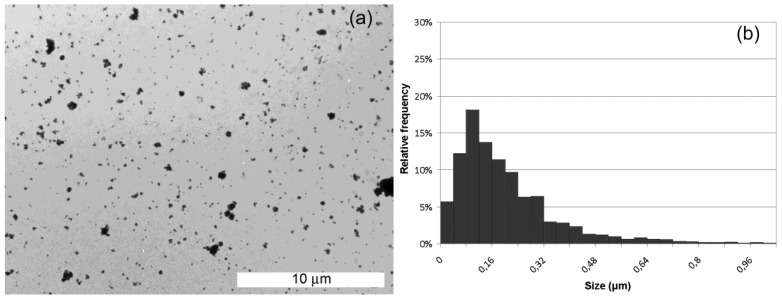
Transmission electron microscopy (TEM) images of crystalline samples milled 20 h (**a**) and particle distribution obtained (**b**).

We have used a high resolution imaging technique to highlight the morphological features of silicon particles produced. Figure 4 (a) shows a TEM microscopy image of cSi-20b particles together with the size distribution (b). The dimensions of the particles have been estimated by an open source software ImageJ for images processing and analysis. First, we have properly thresholded the images, in order to obtain a net contrast between the color of particles and the background; then we have calibrated the Measurement function of the software by using the measure bar reported on each TEM image: in this way we set the ratio between one pixel to some nanometers, which corresponds to the resolution of each measurement. A macro of the software (Set Measurements in the Analyze function) was used to calculate the Feret diameter, which is used to get a value of particle size, since it is distance between two tangents on opposite sides of the particle profile. The Analyze Particles function finally gives a list of the particles individuated and measured that were used to get the histogram. Even if some particles aggregate in clusters, the size distribution can be fitted using a lognormal function, having 50% of particles smaller than 170 ± 16 nm. Particle size statistics have been obtained on more than 200 silicon nanoparticles.

**Figure 5:**
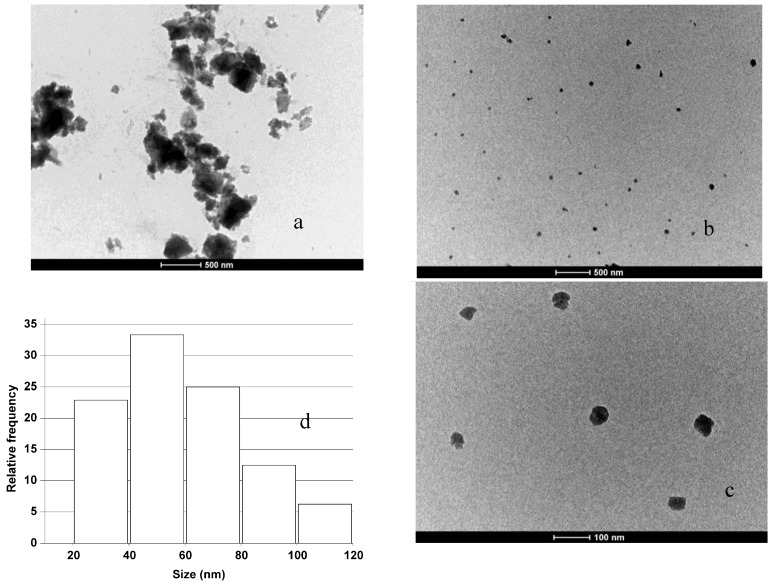
TEM images of the porous samples: (**a**) sample milled 2 h; (**b**) sample after sonication; (**c**) zoomed image of some particles, (**d**) particles size distribution of porous sample milled 2 h.

In Figure 5(a), (b), (c), and (d), the images of nanoparticles obtained starting from porous silicon samples together with the particle size distribution estimated are reported. The nanoparticles showed a strong attitude to aggregate after low power sonication, which produces hundred of nanometer sized clusters clearly visible in 5 (a). More homogeneous samples can be obtained by 10 minutes of high power sonication. In this case, the nanoparticles observed by TEM are well under 100 nanometers and have spheroidal shapes (see Figure 5 (b) and (c)). High power sonication also reduces the aggregation behavior. We have experimentally verified that high power sonication, even for longer times up to 30 min, do not affect crystallite sizes by XRD on treated powders, which are equal to those reported in Figure 2 (b); therefore this sonication process has the only effect of disaggregating the clusters of nanoparticles formed during the milling treatment. In the size distribution of the sample treated for 2 h (pSi-2) we can find that the median, calculated on all the data set available (more than 150 particles in different TEM images acquired in same conditions), is at 55 ± 4 nm, while the average Feret diameter is at 58 ± 4 nm.

The ability to load and release drugs strongly depends on coating the nanoparticles by proper chemical compounds which can be used to graft active molecules on their surface. In our experiments, we have proved the possibility of covalently link on the silicon nanopowders the siloxane APTES, which brings a free -NH_2_ group available to bind every carboxylic terminated biomolecule. 

**Figure 6:**
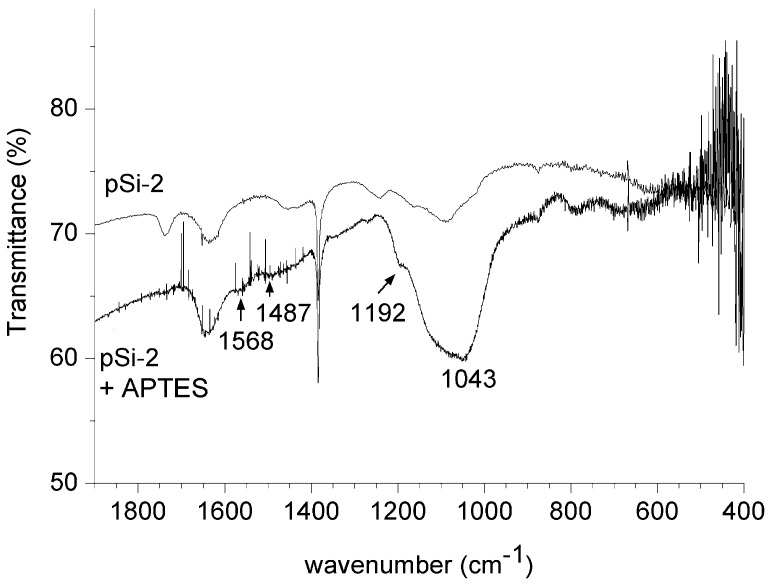
FT-IR spectra for porous sample milled 2 h and the same sample after silanization with APTES.

We have thus wet porous silicon derived samples in order to prove that the milling process can be used for contemporary comminution and chemical functionalization of these particles. As a proof of successful functionalization, the spectra of a powder sample without and after silanization are reported in Figure 6: amino groups peaks appear at 1570 cm^−1^ and 1485 cm^−1^ as well as the non-hydrolyzed ethoxy groups peak at 1192 cm^−1^ [27].

## 4. Conclusions

Nanostructured powders have been produced by a simple but not trivial mechanical technique: the ball milling. The nanopowders have been characterized by means of several analytical methods, each technique provided different information about the structure and chemistry of the samples. Nanometric (<50 nm) powders with high surface area (from 29 up to 100 m^2^/g) have been produced. The milling process, which operates at room temperature and atmospheric pressure, could be appealing from a large-scale fabrication point of view, when industrial milling systems are adopted, and competitive with respect to other synthesis methods. Powders from porous silicon membranes are reduced in nanometric agglomerates in a very short time without destroying the starting silicon skeleton. Chemical passivation of nanoparticles’ surfaces by APTES has also been proved in order to obtain more functionality in biomedical applications.
